# The influence of fixation and cryopreservation of cerebrospinal fluid on antigen expression and cell percentages by flow cytometric analysis

**DOI:** 10.1038/s41598-024-52669-1

**Published:** 2024-01-30

**Authors:** Gabriela Singh, Arjan van Laarhoven, Rozanne Adams, Timothy Dawson Reid, Jill Combrinck, Suzanne van Dorp, Catherine Riou, Nqobile Thango, Johannes Enslin, Stefan Kruger, Anthony Aaron Figaji, Ursula Karin Rohlwink

**Affiliations:** 1 Division of Neurosurgery, Department of Surgery, Neuroscience Institute, University of Cape Town, Cape Town, South Africa; 2 Department of Internal Medicine and Radboud Center of Infectious Diseases, Radboud University Medical Center, Nijmegen, The Netherlands; 3 City of Cape Town, Becton Dickinson (BD) Biosciences, Western Cape, South Africa; 4 South African Tuberculosis Vaccine Initiative (SATVI), Institute of Infectious Disease and Molecular Medicine (IDM), University of Cape Town, Cape Town, South Africa; 5 Department of Hematology, Radboud University Medical Center, Nijmegen, The Netherlands; 6 Institute of Infectious Disease and Molecular Medicine, University of Cape Town, Cape Town, South Africa

**Keywords:** Neuroscience, Diseases of the nervous system

## Abstract

The pauci-cellular nature of cerebrospinal (CSF), particularly ventricular CSF, and the rapid cell death following sampling, incumbers the use of flow cytometric analysis of these samples in the investigation of central nervous system (CNS) pathologies. Developing a method that allows long-term storage and batched analysis of CSF samples without compromising cell integrity is highly desirable in clinical research, given that CSF is often sampled after hours creating logistical difficulties for fresh processing. We examined percentages and relative proportion of peripheral and brain-derived immune cells in cryopreserved and transfix-treated CSF, compared to freshly processed CSF. Cell proportions were more comparable between Fresh and Cryopreserved CSF (mean of differences = 3.19), than between fresh and transfix-treated CSF (mean of differences = 14.82). No significant differences in cell percentages were observed in fresh versus cryopreserved CSF; however significantly lower cell percentages were observed in transfix-treated CSF compared to Fresh CSF [(CD11b^++^ (*p* = 0.01), CD4^+^ (*p* = 0.001), CD8^+^ (*p* = 0.007), NK cells (*p* = 0.04), as well as CD69^+^ activation marker (*p* = 0.001)]. Furthermore, loss of marker expression of various lymphocyte sub-populations were observed in transfix-treated CSF. Cryopreservation is a feasible option for long-term storage of ventricular CSF and allows accurate immunophenotyping of peripheral and brain-derived cell populations by flow cytometry.

## Introduction

The analysis of inflammatory mediators in cerebrospinal fluid (CSF) can provide valuable information pertaining to ongoing disease processes in central nervous system (CNS) conditions^1,2^.This is important for developing countries faced with a high burden of CNS infections, which predominantly affect vulnerable populations like young children, and are accompanied by high morbidity and mortality rates^3–5^. There is evidence which demonstrates that the immune response is compartmentalized to the site of disease in CNS infections, emphasizing the importance of examining site of disease samples such as CSF^2,6,7^. Immunophenotypic characterisation of CSF by flow cytometry has become a popular technique for the study of various neurological conditions^6,8–10^. Unfortunately, CSF samples have low cellularity and are prone to rapid cellular degradation ex vivo, making flow cytometric analysis challenging^11^. It is therefore recommended to immediately process CSF samples for flow cytometric analysis^11^. However, this is often logistically impractical, especially for resource-constrained environments which lack on-site flow cytometers and where sample collection is too unpredictable to book research flow cytometers. Currently, cell stabilizing reagents are used to prolong cell viability in CSF, showing promising results for short-term storage of CSF samples. For example, Transfix has been shown to preserve leukocytes for up to 72 h of storage, with better preservation of lymphocytes than monocytes and granulocytes^12^. However, Transfix immediately fixes cells preventing any downstream functional assays. Advances have been made in developing long-term cryopreservation protocols for specimens including whole blood^13^, stem cells^14^, embryos^15^, and hepatocytes^16^. However, cryopreservation has only recently been attempted in CSF of adult cryptococcal meningitis patients^17,18^. While the results of these studies were promising, cryopreservation was only performed on lumbar CSF, which from a study on paediatric tuberculous meningitis (TBM), has been shown to have a higher cell count compared to ventricular CSF and does not accurately represent pathophysiology at the site of disease in the brain^2^. Furthermore, only lymphocytes were examined, and the studies were conducted exclusively in adults allowing for larger volumes (> 8 ml) of CSF than are commonly collected in paediatric patients. Therefore, the objective of this study was to compare two methods of *long-term* storage of *small volumes* of *ventricular* CSF samples, namely Cryopreservation and Transfix, for immunophenotyping of both *peripheral and brain-derived* (microglia and astrocytes) cell populations using flow cytometry in paediatric CSF infection.

## Methods

### Study cohort and sample collection

This study prospectively recruited paediatric patients admitted to Red Cross War Memorial Children’s Hospital (RCWMCH) between June 2021 and October 2021. Ventricular CSF, and where possible lumbar CSF, and serial samples were collected from patients during clinically indicated neurosurgical procedures. A volume of ≥ 3 ml of CSF was collected from a patient cohort with various CNS infections. An experimental arm was also incorporated into the study. This involved collecting CSF samples from children who required neurosurgical interventions for conditions unrelated to CNS infections. Given that these CSF samples did not contain any cells, they were spiked with peripheral blood mononuclear cells (PBMCs) isolated from the whole blood of healthy adult volunteers.

### Sample processing

A minimum of 3 ml of CSF was required in order to divide the sample across the three protocols (1) “Fresh CSF” serving as the current gold standard; (2) “Transfix-treated CSF” and (3) “Cryopreserved CSF”. Patient CSF samples were processed immediately; whereas experimental CSF samples were left in the fridge for a minimum period of 1 week to ensure the breakdown of any native cells and to ensure that the experimental CSF samples were spiked with a known concentration of PBMCs.

### Patient CSF samples

**Figure 1:**
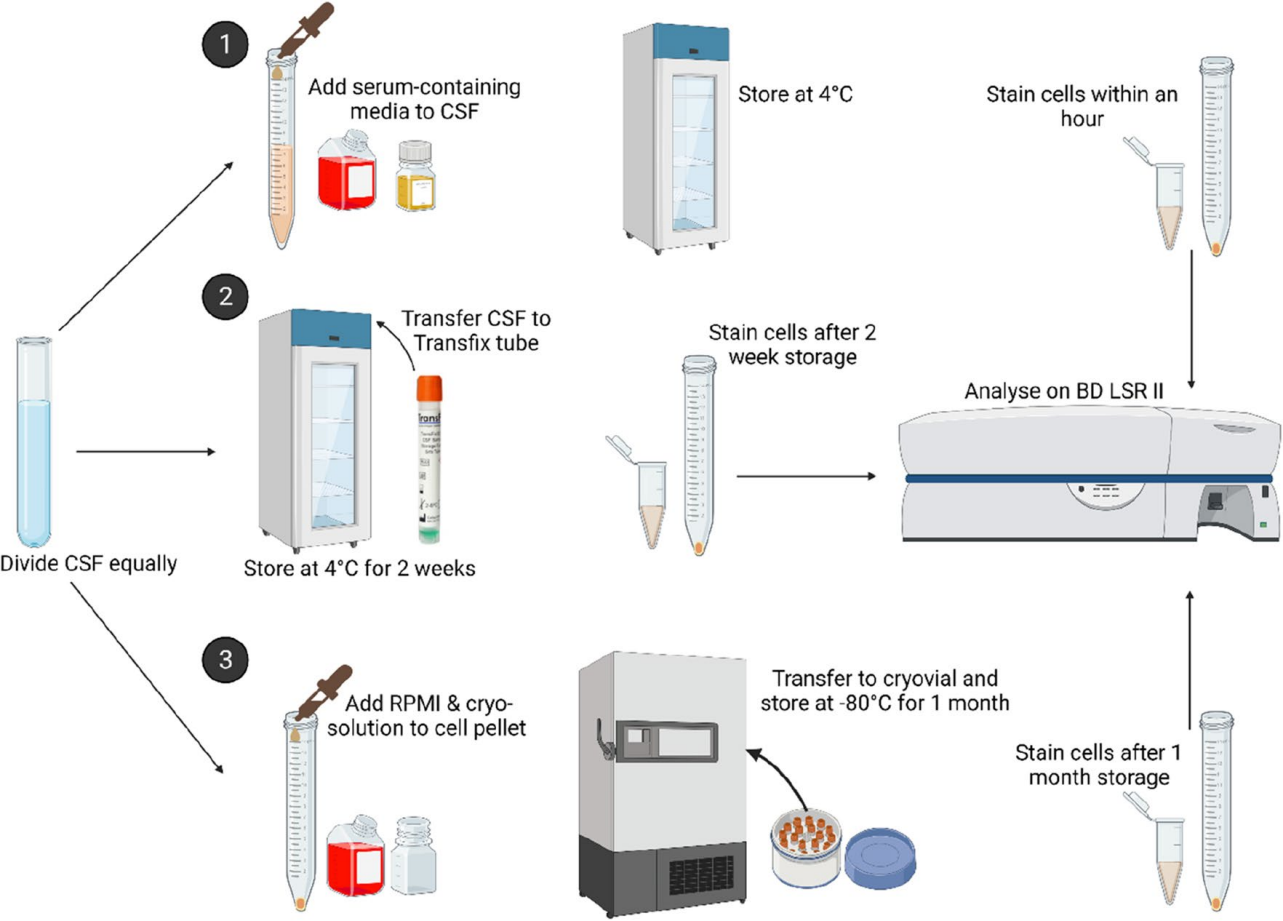
Workflow of CSF sample processing. (**1**) Fresh CSF. (**2**) Transfix-treated CSF. (**3**) Cryopreserved CSF. Created with Biorender.com.

Immediately after withdrawal, the CSF samples were divided equally into the three appropriately labelled sterile tubes. Figure 1 provides a summary of the workflow undertaken.

#### Fresh CSF

CSF was immediately transferred to a tube containing an equal volume of serum-containing media made up of 90% RPMI (Cytiva Hyclone, USA) and 10% heat inactivated foetal bovine serum (FBS) (ThermoFisher, USA), kept at 4 °C and processed within an hour. Briefly, the sample was centrifuged at 300*g* for 5 min at 4 °C with the break turned off. The supernatant was discarded, and the cell pellet was resuspended in 75 µl of antibody cocktail and left to incubate for 30 min in the fridge protected from light. Following incubation, 250 µl of Becton Dickinson (BD) Cytofix/Cytoperm™ buffer was added to the mixture and incubated for a further 20 min in the fridge protected from light. Following the second incubation period, the cell suspension was washed with 250 µl of PermWash buffer (BD, USA). After centrifugation at 510*g* for 5 min, the supernatant was aspirated using a pipette. The cell pellet was resuspended in 100 µl of PermWash buffer and stained with 1.25 µl of GFAP antibody (BD, USA) for 30 min in the fridge protected from light. Following incubation, the cells were washed with 500 µl of PermWash buffer. The supernatant was aspirated, and the cell pellet was resuspended in 150 µl of Flow Staining buffer (R&D, USA), stored at 2–8 °C and acquired within 1 h on the flow cytometer.

#### Transfix-treated CSF

CSF was immediately transferred to a Transfix^®^ tube containing 0.2 ml of Transfix/EDTA (Cytomark, Buckingham, UK) and was stored at 2–8 °C for 2 weeks (as a marker of long-term storage). Given that previous studies stored CSF for a maximum of 72 h, additional storage periods of 24 h and 1 week for sub-group comparisons with the 2 week sample, were included. Following the storage periods, CSF was transferred to a 15 ml Falcon tube, 5 ml of phosphate buffered saline (PBS) (Hyclone laboratory Inc, USA) was used to wash the Transfix^®^ tube and thereafter transferred to the 15 ml Falcon tube. The sample was centrifuged at 300*g* for 5 min at 4 °C with the break turned off. The supernatant was discarded, and the cell pellet was resuspended in 500 µl of PermWash buffer and incubate for 20 min at 4 °C. After the first incubation, the cell suspension was centrifuged at 510*g* for 5 min, the supernatant was aspirated, and the cell pellet was resuspended in 75 µl antibody cocktail for 30 min at 4 °C protected from light. Thereafter, the cells were washed with 500 µl of PermWash buffer. The supernatant was aspirated, and the cell pellet was resuspended in 150 µl of Flow Staining buffer, stored at 2–8 °C and acquired within 1 h on the flow cytometer. A fixation step was not required given that cells were immediately fixed by the Transfix^®^ solution.

#### Cryopreserved CSF

CSF was immediately centrifuged at 300*g* for 5 min with the break turned off. The supernatant was discarded, and the cell pellet was resuspended in 500 µl of cold RPMI. Cryo-solution, made up of 93% of heat inactivated FBS and 7% dimethyl sulfoxide (DMSO) (R&D, USA) was added dropwise to the cell suspension and immediately transferred to a cryovial, placed in a Mr Frosty™ freezing container (Sigma-Aldrich, USA) and stored at − 80 °C for 1 month. Thereafter, cryovials were thawed in a 37 °C water bath by slowly agitating the vial until a block of ice remained. Once partially thawed, 1 ml of room temperature RPMI was immediately added to the vial and transferred to a 15 ml Falcon tube. An additional 1 ml of RPMI was used to wash the cryovial and was also transferred to the 15 ml Falcon tube. Thereafter, the sample was centrifuged at 300*g* for 5 min at 4 °C with the break turned off. The same antibody staining process outlined in the “Fresh” protocol, was used. The cell suspension was stored at 2–8 °C and acquired within 1 h on the flow cytometer.

In summary, there were only minor methodological differences in the way in which the CSF samples were processed across the three different protocols. For instance, in Transfix-treated CSF the fixation step occurred prior to cell surface staining and a viability stain was not included as cells were already fixed once the CSF was added to the Transfix tube.

### Experimental CSF samples

#### Isolation of PBMCs

Briefly, 8 ml of whole blood was collected in vacutainer sodium heparin cell preparation tube (CPT) (BD, USA). The tubes were centrifuged at 1500*g* for 30 min with the break turned off. The plasma was discarded and the layer of PBMCs were carefully isolated and transferred to a 15 ml Falcon tube containing 5 ml of PBS. Following two wash steps with PBS, the cell pellet was resuspended in 1 ml of RPMI and a further 1 ml of cryo-solution, consisting of 20% DMSO and 80% heat inactivated FBS, was added dropwise. The suspension was immediately transferred to cryovials and placed in Mr Frosty™ freezing container for subsequent freezing at − 80 ^∘^C.

#### Spiking of CSF

Firstly, the PBMC cryovials were thawed in a 37 ^∘^C water bath by slowly agitating the vials until a block of ice remained. Thereafter, 1 ml of warmed media, consisting of 90% RPMI and 10% FBS, was added dropwise and transferred to a 50 ml Falcon tube. An additional 19 ml of warmed media was added bringing the final volume to 20 ml. The suspension was centrifuged at 363*g* for 10 min, the supernatant was discarded, and the cells underwent a second wash. Following the 2nd wash, the cell pellet was resuspended in 1 ml of warmed media, an additional 4 ml of media was added. To prevent clumping of cells, 20 µl of DNase (0.24 Kunits/ml) (Sigma-Aldrich, USA) was added and the sample was left to incubate for 2 h. During this time, 10 µl of the cell suspension was aliquoted and sent to the National Health Laboratory Service (NHLS) for a white blood cell count; 90 µl of trypan blue was added to the suspension and cells were enumerated using a Neubauer chamber. Following the 2 h incubation period, the cells were resuspended in media to have a final concentration of 1 × 10^7^ cells/ml. The CSF sample was spiked with a known concentration of cells and the three protocols were immediately carried out as outlined above.

### Flow cytometry

Cells were stained using a 16-colour antibody panel (Table 1) for which optimal antibody titres were calculated prior to use (Supplementary Table 1). Fluorescence-minus-one controls were used to accurately identify and gate populations of interest (refer to Supplementary Fig. 1 for gating strategy). Sample acquisition was carried out on a BD LSR II flow cytometer, 50,000 events were recorded for patient samples and 1,000,000 events were recorded for experimental samples given the higher cell count. Immunophenotyping of major cell populations of interest in this study, are listed in Table 2. Major cell populations were identified using distinct lineage markers. The choice of sub-populations investigated were based on data reported in adult TBM and paediatric studies of disseminated tuberculosis (TB)^6,19^.

**Table 1:** List of fluorochrome-conjugated antibodies.

Laser configuration		Fluorochrome	Marker	Clone	Specificity
Filter 1	Filter 2				
740 LP	780/60 BP	APC-H7	CD3	SK7	T cells
505 LP	515/20 BP	BV510	CD4	SK3	T cells
685 LP	710/50 BP	PerCP-Cy5.5	CD8	SK1	T cells
680 LP	710/50 BP	APC-R700	CD69	FN50	Activation
505 LP	515/20 BP	BB515	CD19	HIB19	B cells
585 LP	605/40 BP	BV605	CD14	M5E2	Monocytes
740 LP	780/40 BP	PE-Cy7	CD16	3G8	NK & Monocytes
770 LP	785/60 BP	BV786	HLA-DR	G46-6	Activation
N/A	670/30 BP	APC	CD56	B159	NK cells
635 LP	660/20 BP	PE-Cy5	CD161	DX12	MAIT cells
685 LP	710/40 BP	BV711	Vα7.2	OF-5A12	MAIT cells
630 LP	655/40 BP	BV650	γδ TCR	B1	γδ cells
595 LP	610/20 BP	PE-CF594	CD11b	ICRF44	Monocytes/macrophages and microglia
N/A	450/50 BP	V450	CD45	HI30	Early differentiation
N/A	575/25 BP	PE	GFAP	1B4	Astrocyte activation
550 LP	572/36 BP	BV570	Live/dead	None	Viability

*APC* allophycocyanin hillite 7, *BB* brilliant blue 515, *BD* Becton Dickinson, *BV* brilliant violet, *CD* cluster of differentiation, *GFAP* glial fibrillary acidic protein, *HLA-DR* human leukocyte antigen-DR isotype, *MAIT* mucosal associated invariant T cell, *NK* natural killer, *PE* phycoerythrin, *PerCP* peridinin-chlorophyll-protein complex. All antibodies were purchased from BD Biosciences.

**Table 2:** Immunophenotypic characterisation of cell populations.

Cell population	Immunophenotypic definition
Leukocytes	CD45^+^
Microglia	CD45^+^ CD11b^++^
Non-microglia	CD45^+^ CD11b^+^
T cells	CD3^+^
T-helper cells	CD3^+^CD4^+^
Cytotoxic T cells	CD3^+^CD8^+^
MAIT cells	CD3^+^ CD161^+^ Vα7.2^+^
B cells	CD3^-^ CD19^+^
NK cells	CD16^+^ CD56^+^
Classical monocytes	CD14^+^ CD16^-^
Non classical monocytes	CD14^-^ CD16^+^
Astrocytes	CD45^-^ GFAP^+^

*CD* cluster of differentiation, *GFAP* glial fibrillary acidic protein, *MAIT* mucosal associated invariant T cell, *NK* natural killer.

### Data and statistical analysis

Exported flow cytometry standard (FCS) files were analysed, and gating was performed on FlowJo software (version 10.7.2). Statistical analyses were performed using IBM SPSS Statistics version 27 (IBM Corp, Armonk, New York, USA). Descriptive statistics were performed to determine the distribution of the data, abnormally distributed data were reported as median and interquartile range or minimum–maximum range. Friedman test was used to compare percentages of different cell populations and median fluorescent intensity (MFI) values between the methods. Bland–Altman test was used to evaluate the level of agreement between the Fresh and Cryopreservation, and Fresh and Transfix methods in patient and experimental samples. A Spearman’s correlation was also performed on cell percentages between Fresh and the Cryopreservation/Transfix methods. The designated level of statistical significance was set at 0.05.

### Ethical approval

Informed consent was obtained from the parents and/or guardians. This study received the necessary approval by the Department of Surgery Research Committee (2020/073), Human Research Ethics Committee of the University of Cape Town (HREC 368/2020) and the Research Review Committee of RCWMCH (RCC 247/WC_202010_049); all methods were carried out in accordance with relevant guidelines and regulations. Assent was requested in children over the age of 8 years with a Glasgow Coma Scale score of 15 (fully conscious). All research was performed in accordance with the Declaration of Helsinki.

## Results

A total of 30 CSF samples were collected, 10 from the patient cohort and 20 from the experimental cohort. The patient cohort comprised three patients with clinically diagnosed TBM and three with bacterial meningitis. One patient sample was excluded due to an abundance of pus, resulting in poor cell isolation and therefore only 29 CSF samples were included for analyses. Table 3 provides the demographic and clinical characteristics of the study cohort.

**Table 3:** Demographic and clinical characteristics of study cohort.

Variable	Patient cohort	Experimental cohort
Number		
Patients	5	13
Samples	9	20
Lumbar CSF	2	0
Ventricular CSF	7	20
Demographics		
Age (years)	1.6 (0.5–1.7)	1.6 (0.6–9.3)
Sex (male)	3 (60)	7 (54)
Clinical characteristics		
Pathology		
TBM	3	
Bacterial meningitis	3	
Total WBC count (cells/µl)	127 (11–535)^a^	1 million^b^

Values reported as median (interquartile range), number (percent).

*CSF* cerebrospinal fluid, *WBC* white blood cell count.

^a^White blood cell (WBC) counts is the sum of lymphocytes and polymorphonuclear cells.: the NHLS only reports lymphocytes and polymorphonuclear cells, other cell types are not routinely reported.

^b^Cell count (cells/ml) after spiking CSF of experimental samples.

### Comparison of cell percentages across methods

All statistically significant differences for the comparison between methods are displayed in Table 4. Summary statistics of cell percentages across methods for patient and experimental samples are included in Supplementary Tables 2 and 3; and the distribution of data of select cell populations are shown in Supplementary Fig. 2.

**Table 4:**
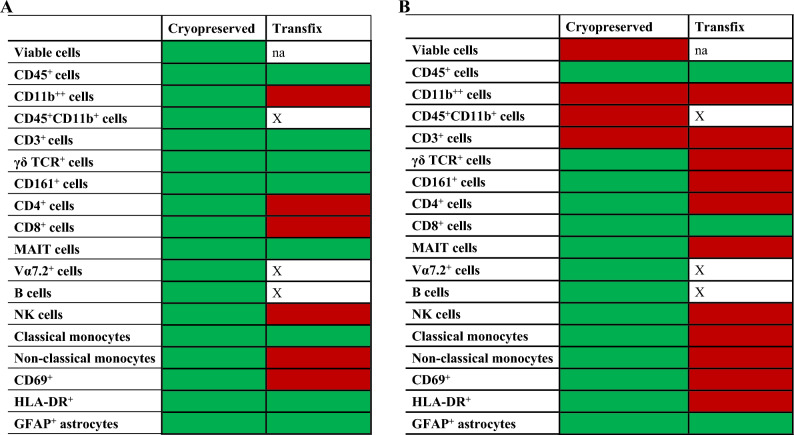
Comparison of cell percentages across methods in (**A**) patient samples (n = 9) and (**B**) experimental samples (n = 20).

Cell percentages were compared between fresh vs cryopreservation, and fresh vs transfix methods. Green boxes signify no statistically significant difference, whereas red boxes signify statistically significant differences relative to fresh. Boxes demarcated with “X” represent cell populations that could not be clearly distinguished from other populations and could not be included in the analysis.

*CD* cluster of differentiation, *HLA-DR* human leukocyte antigen-DR, *GFAP* glial fibrillary acidic protein, *TCR* T cell receptor.

#### Fresh versus cryopreservation

When comparing fresh to the cryopreserved samples, no significant differences were found for any of the cell populations or activation markers in patient samples (Supplementary Table 2). However, for the experimental samples, significant differences were found in cell viability (47.15% vs 43.3%, *p* = 0.05), CD11b^++^ (9.34% vs 14.35%, *p* = 0.01), CD45^+^CD11b^+^ (91.25% vs 86.25%, *p* = 0.01) and CD3^+^ cells (72.75% vs 69.3%, *p* = 0.02) between fresh and cryopreserved methods (Supplementary Table 3). In some cases, fresh had higher percentages, in some cases Cryopreservation had higher percentages—Supplementary Fig. 3.

#### Fresh versus transfix

**Figure 2:**
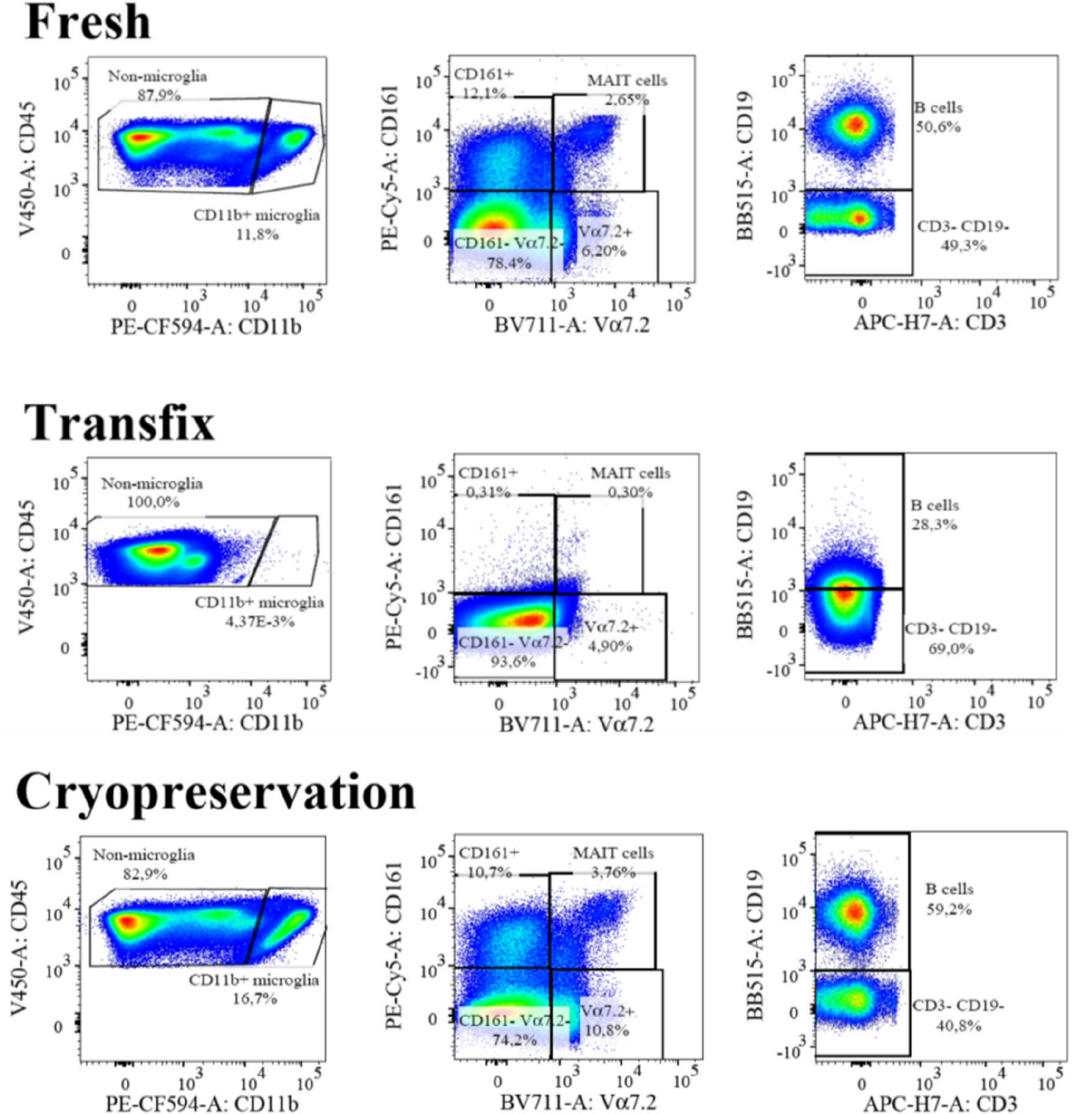
Example of cell populations that were not clearly distinguishable in transfix compared to fresh and cryopreservation methods. CD45^+^CD11b^+^ (non-microglia), Vα7.2^+^, and B cells could not be clearly defined in transfix. Data from same sample.

For patient CSF samples, significantly higher percentages of CD11b^++^ (2.64% vs 0.028%, *p* = 0.01), CD4^+^ (69.1% vs 47.7%, *p* = 0*.*001), CD8^+^ (19.6% vs 12%, *p* = 0*.*007), NK cells (62.3% vs 18%, *p* = 0*.*004), non-classical monocytes (34.2% vs 0.07, *p* = 0*.*03) as well as CD69^+^ activation marker (41.2% vs 3.25%, *p* = 0*.*001) were observed in the Fresh method compared to Transfix (Supplementary Table 2). In experimental CSF samples, significantly higher percentages of CD11b^++^ (9.34% vs 0.021%, *p* < 0*.*001), CD3^+^ (72.75% vs 60.7%, *p* < 0*.*001), γδ TCR^+^ (0.48% vs 1.95%, *p* = 0.01), CD4^+^ (87.1% vs 47.6%, *p* < 0*.*001), MAIT (6.38% vs 1.39%, *p* = 0*.*007), NK cells (32.5% vs 3.64%, *p* < 0*.*001), classical (1.12% vs 7.75%, *p* < 0*.*001) and non-classical monocytes (58.6% vs 0.69%, *p* < 0*.*001), as well as CD69^+^ (0.98% vs 0.02%, *p* < 0*.*001) and HLA-DR^+^ activation markers (2.21% vs 0.91%, *p* < 0*.*001), were found in the Fresh method compared to Transfix (Supplementary Table 3). Certain cell populations such CD45^+^CD11b^+^, Vα7.2^+^, and B cells could not be clearly distinguished from other cell populations in Transfix-treated CSF (Fig. 2). Thus, cell percentages for these sub-populations were not included in the analyses. This observation was apparent in both patient and experimental CSF samples.

### Assessing the level of agreement between methods

**Figure 3:**
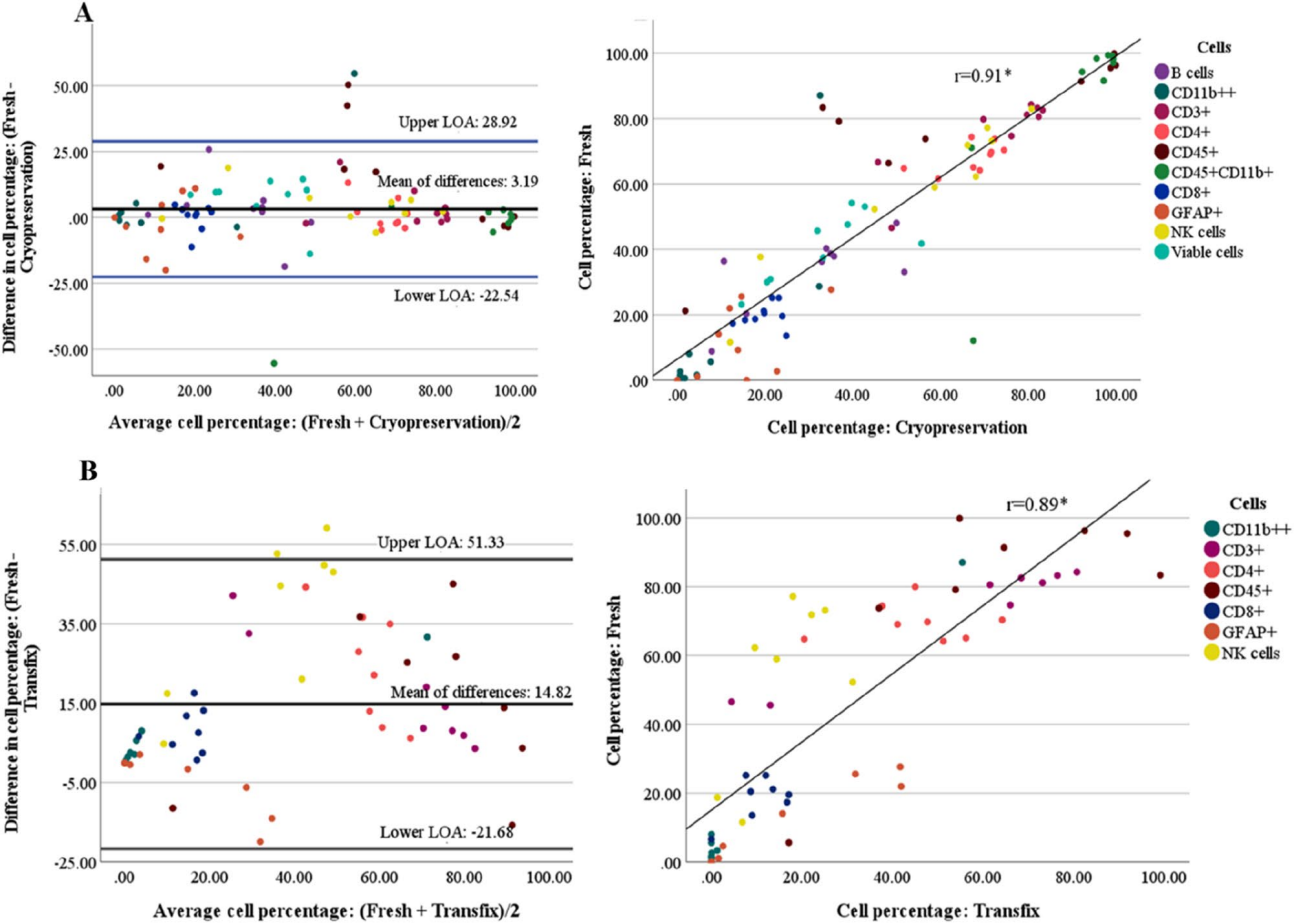
Assessing the level of agreement between methods in patient samples (n = 9). Bland–Altman plot of the differences in cell percentages between methods vs the average of the two methods. (**A**) Fresh vs cryopreservation. The bias (3.19) is represented by the mean of differences, with the upper-and lower levels of agreement (LOA) displayed as horizontal lines on the plot. A significant positive correlation (r = 0.91) between methods was observed. (**B**) Fresh vs transfix. The bias (14.82) is represented by the mean of differences, with the upper-and lower levels of agreement (LOA) displayed as horizontal lines on the plot. A significant positive correlation (r = 0.89) between methods were observed. The cell types assessed are colour-coded as represented by the legend*.*

**Figure 4:**
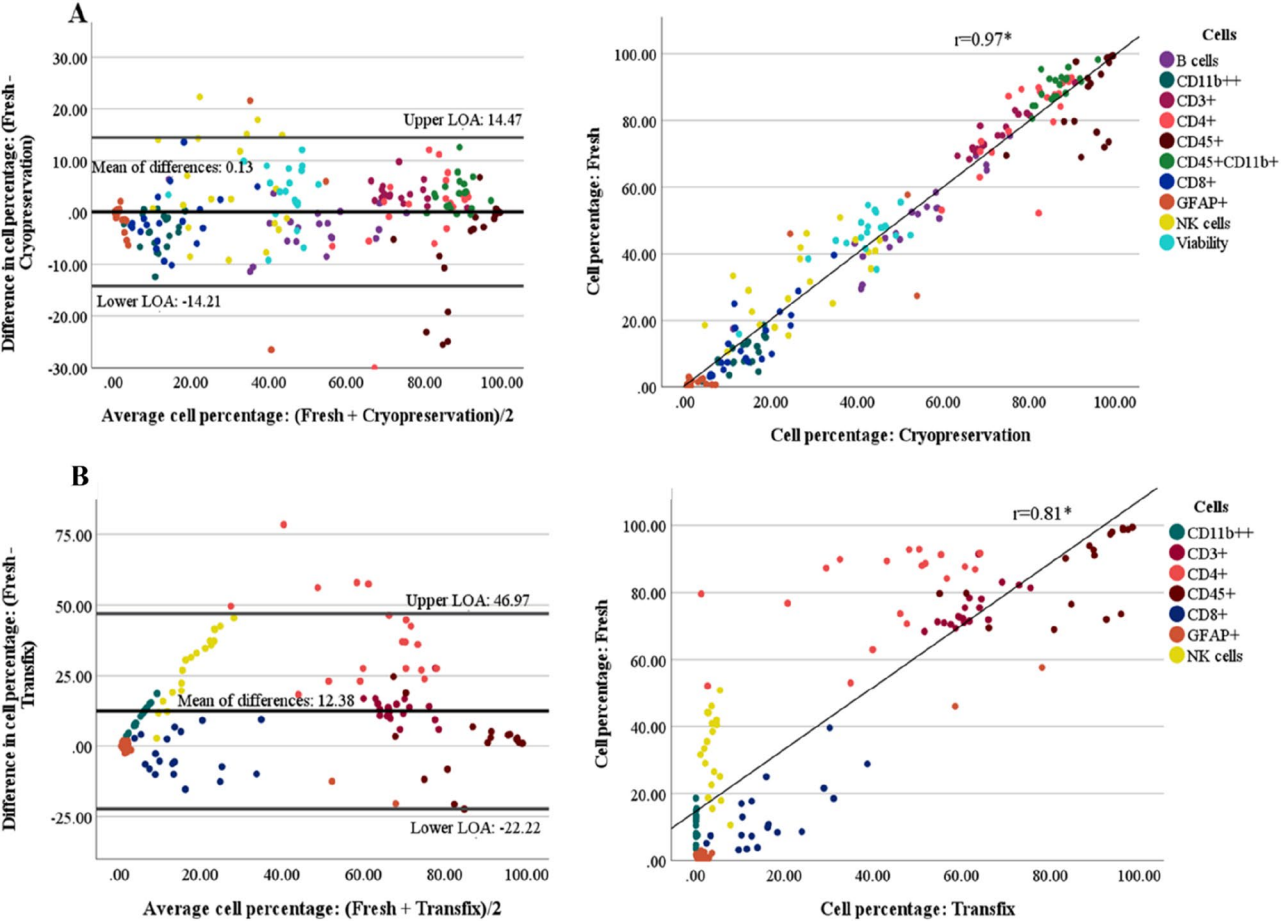
Assessing the level of agreement between methods in experimental samples (n = 20). Bland–Altman plot of the cell percentage differences between methods vs the average of the two methods. (**A**) Fresh vs cryopreservation. The bias (0.13) is represented by the mean of differences, with the upper-and lower levels of agreement (LOA) displayed as horizontal lines on the plot. A significant positive correlation (r = 0.97) between methods were observed. (**B**) Fresh vs transfix. The bias (12.38) is represented by the mean of differences, with the upper-and lower levels of agreement (LOA) displayed as horizontal lines on the plot. A significant positive correlation (r = 0.81) between methods were observed. The cell types assessed are colour-coded as represented by the legend.

In the fresh vs cryopreservation method comparison, all major cell types were included in the analyses; whereas in the Fresh vs Transfix method comparison, only cell types that could be clearly defined during flow gating were included (as described above). Results for patient and experimental samples are illustrated in Figs. 3 and 4, respectively.

In patient samples, the estimated level of agreement was greater between fresh and cryopreservation methods (mean of differences = 3.19) than between fresh and transfix methods (mean of differences = 14.82) (Fig. 3A,B, left panel). This was further emphasized by the range of the limits of agreement, with fresh vs cryopreservation having a smaller range (− 22.54 to 28.92) compared to fresh vs transfix methods (− 21.68 to 51.33) (Fig. 3A,B, left panel). Both Fresh vs Cryopreservation and Fresh vs Transfix methods had a significant positive correlation of r = 0.91 (*p* < 0*.*001) and r = 0.81 (*p* < 0*.*001), respectively (Fig. 3A,B, right panel).

In experimental samples, similarly, fresh and cryopreservation methods demonstrated a greater level of agreement compared to fresh and transfix methods, with the mean of differences being 0.13 and 12.38, respectively (Fig. 4A,B, left panel). The range of the limits of agreement were also smaller in fresh and cryopreservation (− 14.21 to 14.47) compared to fresh and transfix (− 22.22 to 46.97) (Fig. 4A,B, left panel). Both fresh vs cryopreservation and fresh vs transfix methods had a significant positive correlation of r = 0.97 (*p* < 0*.*001) and r = 0.81 (*p* < 0*.*001), respectively (Fig. 4A,B, right panel).

### Duration of storage of transfix-treated experimental CSF samples

Compared to the fresh method, CSF stored in transfix for 24 h yielded significantly higher CD45^+^ (*p* = 0*.*05), CD11b^++^ (*p* = 0*.*046), CD161^+^ (*p* = 0*.*046), and MAIT (*p* = 0*.*05) cell percentages (Supplementary Table 4). However, in comparison to 1 week and 2 week storage in transfix, the fresh method still yielded significantly higher cell percentages. Additionally, for the remaining cell populations and activation markers, the fresh method yielded significantly higher percentages compared to 24 h, 1 week, and 2 week storage in transfix (see Supplementary Table 5 for *p* values).

### Influence on fluorescent signal

#### Fresh versus cryopreservation

**Figure 5:**
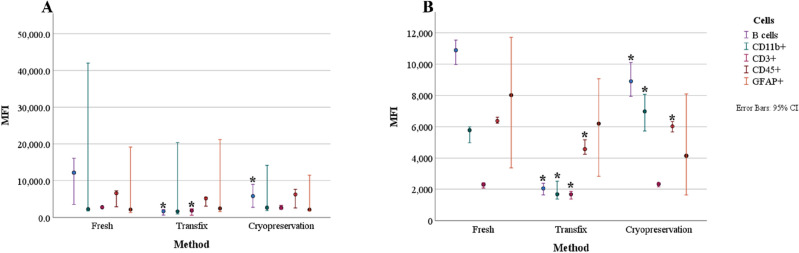
Difference in median fluorescent intensities (MFI) of cell markers between methods. (**A**) Patient samples. (**B**) Experimental samples. CD19^+^, CD11b^+^, CD3^+^, CD45^+^, and GFAP^+^ cell markers were assessed. Median MFI values are plotted on the y-axis, with 95% confidence intervals (CI) represented by the error bars. Statistically significant differences in comparison to fresh are illustrated with an asterisk (*p < 0.05).

Significantly higher MFIs for CD19^+^ (*p* = 0*.*024) were observed for the fresh method in patient samples (Fig. 5A). In experimental samples, the fresh method yielded significantly higher MFIs for CD45^+^ (*p* = 0*.*009) and CD19^+^ (0*.*001), whereas the Cryopreservation method yielded significantly higher MFI for CD11b^+^ (*p* = 0*.*021) (Fig. 5B).

#### Fresh versus transfix

In patient samples, MFI values for CD19^+^ (*p* = 0.01) and CD3^+^ (*p* = 0*.*001) markers were significantly higher in the Fresh method (Fig. 5A). Similar findings were also observed for MFI values of CD19^+^ (*p* = 0*.*00) and CD3^+^ (*p* = 0*.*00) markers in experimental samples. In addition, CD45^+^ (*p* = 0*.*009) and CD11b^+^ (*p* = 0*.*00) MFIs were significantly higher for the Fresh method in experimental samples (Fig. 5B).

## Discussion

In this study, the long-term storage effects of cryopreservation and transfix on peripheral and brain-derived immune cell percentages and proportions in prospectively collected CSF samples were assessed by flow cytometric analysis and compared to freshly processed CSF (current gold standard). Unlike previous studies which only cryopreserved lumbar CSF from adults^17,18^, our study was the first to attempt long-term cryopreservation of ventricular CSF obtained from children with CNS infections. Ventricular CSF is pauci-cellular compared to lumbar CSF, but more demonstrative of ongoing disease processes at the site of disease^2^. In keeping with this observation, this is the first study to detect brain-derived cells (microglia and astrocytes) in cryopreserved CSF. Given that the role resident brain cells play in pathology is largely poorly understood, these findings are promising for future studies focused on elucidating site-of-disease pathophysiological processes.

Significant differences in viability, CD11b^++^, CD45^+^CD11b^+^, and CD3^+^ cell percentages between Fresh and Cryopreservation methods were only observed in experimental samples and not patient samples. Although significant, these differences were small, and, in some cases, median values were higher in Cryopreserved CSF than in fresh CSF. Furthermore, these cell populations could still be accurately and clearly defined in cryopreserved CSF. Overall, the Cryopreservation method demonstrated highly comparable results to the fresh method in both patient and experimental samples with small variation in the results and little bias in the variability (respective mean differences of 3.19 and 0.13). In combination these results suggest that cryopreservation may serve as an acceptable alternative method to fresh processing of samples.

Transfix yielded significantly lower percentages of many cell populations in both patient and experimental samples, certain sub-populations were no longer identifiable, and the Bland Altman analysis suggested large variation in the results with clear biases in variation for specific cell types from the transfix method. This did appear to be related to the duration of storage; the maximum storage periods used in previous studies were 18–72 hours^12,20^, and our 24 h storage of CSF in Transfix yielded significantly higher percentages of CD45^+^, CD11b^++^, CD161^+^, and MAIT cells relative to Fresh CSF. This is similar to the findings of De Jongste et al. who reported significantly higher absolute counts of lymphocytes in transfix-treated CSF after 18 h of storage compared to CSF collected in serum-containing media^12^. Nonetheless, the Fresh method still yielded significantly higher percentages compared to the 24 h storage time for CD45^+^CD11b^+^ (non-microglia), and B cells, and clear separation of these cells were not possible after 1 week of storage in transfix. These results suggest that transfix, even following 24 h storage, may not be a suitable cell stabilizing agent for markers of major cell populations such as B cells, NK cells, and monocytes, and minor cell populations including MAIT cells or activation markers. Additionally, due to the fixative in the Transfix, no additional functional assays can be performed on CSF samples. The use of alternative fluorochrome-conjugated antibodies may have produced different results, however, the combination of fluorochromes used in this study resulted in minimal spill-over issues in such a large panel.

Significantly lower MFI values were observed in cryopreserved and transfix-treated CSF, however, the difference in mean ranks between the two separate comparisons was greater in fresh vs transfix than in fresh vs cryopreservation methods. The lower MFI could be due to the length of storage time of cells in cryo-solution (1 month) and transfix (2 weeks), the freeze–thaw cycle in the cryopreservation method, and the additional wash steps included in both methods which may have caused a loss of epitopes on the cell. Fixation with formaldehyde, although advantageous, can mask epitopes through crosslinking and make it more difficult for antibodies to bind to their target sites. All three methods included a fixation step, but as shown in a previous study^21^, the timing of this step appears to be of significance. The fixation step in both fresh and cryopreservation methods was included after antibody staining, the difference in mean ranks was smaller between these methods; whereas in transfix, fixation occurs immediately once CSF is added to the tube, and a greater difference in mean ranks was observed. Therefore, fixation prior to antibody staining may reduce available epitopes on cell surfaces. The loss of fluorescent signal in cryopreserved CSF did not, however, appear to negatively impact on determining cell proportions and cell populations could be easily distinguished, unlike in transfix-treated CSF. Similarly, permeabilizing the cells, which is required for intracellular staining, did not negatively impact the ability to immunophenotype cells in CSF.

Low percentages of viable cells were observed in freshly processed and cryopreserved CSF in both patient and experimental samples. This was an unexpected finding, a possible explanation for this in patient samples could be the presence of exotoxins within CSF. Most exotoxins are polypeptides produced by pathogenic Gram-positive and Gram-negative bacteria^22^ that are responsible for inducing apoptotic and- or necrotic cell death of host immune cells during infection^23^. Thus, the low cell viability observed in patient samples may be a result of cells having undergone cell death prior to CSF sampling and may therefore, not be a reflection of systematic error introduced by the processing methods. The addition of streptomycin/penicillin to media and cryo-solution may improve cell viability, these antibiotics were included in the serum-containing media used by De Graaf et al. who reported improved viability in CSF cells^11^. Low cell viability in the experimental samples may have been a result of the multiple freeze thaw cycles; the first occurred when the PBMCs were isolated and cryopreserved for subsequent spiking, and the second was during the cryopreservation method.

Low percentages of GFAP^+^ astrocytes were quantified in experimental CSF samples, which was unexpected given that these samples were spiked with peripheral leukocytes only. Although the CSF samples were obtained from patients with non-infectious CNS conditions, 85% of this cohort had hydrocephalus. Hydrocephalus is a condition which develops from the excessive accumulation of CSF in the ventricles which increases the intracranial pressure^24^. Significantly elevated levels of GFAP have been previously found in the CSF of patients with hydrocephalus, which may be indicative of reactive astrogliosis in response to raised pressure, especially affecting the ependyma (tissue surrounding the ventricles)^25^. The presence of GFAP^+^ astrocytes in our experimental CSF samples could, therefore, be a result of the brain’s response to hydrocephalus.

Results in experimental samples demonstrated greater differences between CSF processing methods relative to patient samples, possibly because of smaller patient sample numbers, which may have masked significant differences in cell percentages, and because PBMCs underwent two freeze–thaw cycles which may have contributed to cell death.

While this study is the first to report the effects of cryopreservation on CSF cells, there are certain limiting factors. The small sample size for patient samples may have resulted in significant differences in cell percentages being missed. Experimental samples represent an artificial environment and the PBMCs used to spike the CSF were drawn from healthy adult volunteers, these cells may differ from paediatric patients that have an ongoing infectious processes. Absolute numbers of cell populations were not assessed using true count beads in this study; however, the aim of this study was to assess whether cell proportions and phenotypes could be accurately identified following cryopreservation or transfix storage. CD11b was selected as a marker for microglia but it is not specific to brain-derived microglia, and is also expressed by lymphocytes, monocytes, and neutrophils, likely explaining the presence of CD11b+ cells in experimental samples. A more specific marker, such as transmembrane protein (TMEM) 119, could be considered for future studies. Furthermore, a loss in neutrophils was observed in cryopreserved CSF, therefore subsequent gating was not performed.

In conclusion, this study shows that cryopreservation is an acceptable alternative where fresh CSF processing is not feasible. Downstream functional assays should be feasible in cryopreserved CSF, unlike transfix, seeing as cells are isolated from the supernatant first before the addition of cryo-solution. This capacity increases translational research potential, particularly in countries faced with high disease burden of CNS infections and limited laboratory resources and may be extended to a broad spectrum of paediatric CNS conditions (including malignancies and auto-immunity) where the application of flow cytometry is currently limited by resource constraints and low cell counts. This method also allows CSF samples to be easily transported between sites in multi-centre studies and shared between research units, promoting collaborations between institutions. Interesting future research could include examining whether this method could work equally well for (1) other cell populations such as plasma cells, which were not included in this current study; and (2) adult CSF samples.

### Supplementary Information

Supplementary Information.

## Data Availability

The datasets generated during the current study will be made available on the University of Cape Town’s publicly available data repository (https://zivahub.uct.ac.za/).
