# Supplementary material for: The influence of fixation and cryopreservation of cerebrospinal fluid on antigen expression and cell percentages by flow cytometric analysis
Source: Sci Rep. 2024 Jan 30;14:2463. doi: 10.1038/s41598-024-52669-1 (PMC10827736; doi:10.1038/s41598-024-52669-1)
Supplement: Supplementary file 1 — Supplementary Information. [file 41598_2024_52669_MOESM1_ESM.docx]

Supplementary Tables & Figures to

***“The Influence of Fixation and Cryopreservation of Cerebrospinal Fluid on Antigen Expression and Cell Percentages by Flow Cytometric Analysis”***

**Gabriela Singh^1*^, Arjan van Laarhoven^2^, Rozanne Adams^3^, Timothy Dawson Reid^4^, Jill Combrinck^1^, Suzanne van Dorp^5^, Catherine Riou^6^, Nqobile Thango^1^, Johannes Enslin^1^, Stefan Kruger^1^, Anthony Aaron Figaji^1^, Ursula Karin Rohlwink ^1,7^**

**Supplementary Table 1: Calculated antibody titres**

| Antibody | MFI (-) | rSD (-) | MFI (+) | Stain index^a^ | Dilution factor | Volume (µl) |
| --- | --- | --- | --- | --- | --- | --- |
| CD3 APC-H7 | 70 | 101 | 6429 | 31.5 | 1:120 | 1.25 |
| CD4 BV510 | 280 | 248 | 11388 | 22.4 | 1:120 | 1.25 |
| CD8 PerCP-Cy5.5 | 59 | 42 | 5154 | 60.7 | 1:120 | 1.25 |
| CD19 BB515 | 66 | 77 | 6265 | 40.3 | 1:480 | 0.31 |
| CD14 BV605 | 26 | 24 | 721 | 14.5 | 1:60 | 2.5 |
| CD16 PE-Cy7 | 12 | 97 | 859 | 7.3 | 1:120 | 1.25 |
| Vα7.2 BV711 | 96 | 179 | 1732 | 4.6 | 1:120 | 1.25 |
| γδ TCR BV 650 | 60 | 89 | 1035 | 5.5 | 1:120 | 1.25 |
| CD45 V450 | 152 | 113 | 7194 | 31.2 | 1:120 | 1.25 |
| HLA-DR BV786 | 66 | 137 | 12351 | 44.8 | 1:120 | 1.25 |
| CD69 APC R700 | 16 | 30 | 219 | 3.38 | 1:120 | 1.25 |
| CD56 APC^b^ | 9 | 18 | 451 | 12.28 | 1:60 | 5 |
| CD161 PE-Cy5^b^ | 34 | 50 | 1065 | 10.31 | 1:60 | 5 |
| GFAP PE | 462 | 308 | 2388 | 296604 | 1:120 | 1.25 |
| CD11b PE-CF594 | 140 | 177 | 30389 | 2677037 | 1:120 | 1.25 |
| Live/Dead BV570^c^ | 28 | 66 | 2089 | 15.6 | 1:960 | 0.15 |

Serial dilutions were made for antibodies using the manufacturers’ recommended volume as starting point. All manufacturers’ recommended volumes were 5µl, unless stated otherwise. The stain index was calculated to obtain the optimal antibody titre which was used to prepare the antibody cocktail. Note: only the dilution factors resulting in the highest stain index are included in the Table. Abbreviations: APC-H, allophycocyanin hillite; BB, brilliant blue; BV, brilliant violet; CD, cluster of differentiation; GFAP, glial fibrillary acidic protein; HLA-DR, human leukocyte antigen-DR isotype; MAIT, mucosal associated invariant T cell; MFI, mean fluorescent intensity; NK, natural killer; PE, phycoerythrin; PerCP, peridinin-chlorophyll-protein complex; rSD, robust standard deviation.

**^a^**$Stain Index= \frac{MFI \left( + \right) population-MFI \left( - \right)population}{2 \times rSD \left( - \right) population}$

**^b^** The manufacturers’ recommended volume was 10 µl.

**^c^** The manufacturers’ recommended volume was 1 µl.

**Supplementary Table 2: Summary statistics of cell percentages across methods for patient samples**

|  | Fresh | Transfix | Cryopreservation | *P* value | *P* value |
| --- | --- | --- | --- | --- | --- |
| *Patient samples* |  |  |  |  |  |
| Viable cells | 41.8 (30.9-47.6) |  | 33.2 (21.2-39.7) | *na* | *0.481* |
| CD45^+^ cells | 83.4 (73.8-95.5) | 64.6 (54.35-87.1) | 56.5 (36.8-98.7) | *0.41* | *0.63* |
| CD11b^++^ cells | 2.64 (1.53-8.06) | 0.028 (0.01-0.05)* | 2.68 (0.86-7.58) | *0.01* | *0.74* |
| CD45^+^CD11b^+^ cells | 97.1 (91.6-98.4) |  | 97.1 (92.2-99.1) | *na* | *0.85* |
| CD3^+^ cells | 80.6 (74.7-82.6) | 68.4 (63.75-74.75) | 79.6 (69.7-82) | *0.08* | *0.53* |
| γδ TCR^+^ cells | 1.51 (1.32-2.8) | 3.14 (1.85-10.06) | 1.25 (0.83-1.54) | *0.07* | *0.25* |
| CD161^+^ cells | 25.1 (21.5-28.7) | 6.22 (5.32-20.99) | 23.1 (21.9-27.5) | *0.08* | *0.80* |
| CD4^+^ cells | 69.1 (648-70.4) | 47.7 (39.4-53.7)* | 68.9 (67-71.5) | *0.001* | *0.91* |
| CD8^+^ cells | 19.6 (18.4-21.2) | 12 (8.83-15.15)* | 19.8 (17.7-23.1) | *0.007* | *1.0* |
| MAIT cells | 0.88 (0.63-1.71) | 0.45 (0.22-3.96) | 0.83 (0.71-1.02) | *0.90* | *0.97* |
| Vα7.2^+^ cells | 8.46 (6.17-12.4) |  | 6.74 (6.4-7.32) | *na* | *0.97* |
| B cells | 36.4 (33.1-38.7) |  | 33.9 (15.7-35.7) | *na* | *0.28* |
| NK cells | 62.3 (52.3-73.2) | 18 (11-23.6)* | 66.1 (44.9-70.6) | *0.004* | *0.63* |
| Classical monocytes | 2.99 (0.44-4.96) | 4.91 (3.5-6.73) | 0.96 (0.45-2.68) | *0.08* | *0.85* |
| Non-classical monocytes | 34.2 (24.9-43.2) | 0.07 (0.02-0.60)* | 37.2 (17.3-39.1) | *0.03* | *0.63* |
| CD69^+^ | 41.2 (38.28-43) | 3.25 (1.86-12.9)* | 42.5 (32.3-48.6) | *0.001* | *0.53* |
| HLA-DR^+^ | 21.7 (17.9-28.4) | 9.84 (7.51-25.02) | 24.9 (23.1-35.9) | *0.07* | *0.17* |
| GFAP^+^ astrocytes | 9.24 (1.09-22) | 15.7 (0.83-36.75) | 13.8 (9.4-15.8) | *0.63* | *0.58* |

Note: Transfix data excluded for CD45^+^CD11b^+^, Vα7.2^+^, and B cells. Values reported as median (interquartile range). Abbreviations: CD, cluster of differentiation; GFAP, glial fibrillary acidic protein; HLA-DR, human leukocyte antigen-DR; MAIT, mucosal associated invariant T cell; NK, natural killer; TCR, T cell receptor. Statistically significant results compared to the Fresh method are illustrated with an asterisk (*, p<0.05).

**Supplementary Table 3: Summary statistics of cell percentages across methods for experimental samples**

|  | Fresh | Transfix | Cryopreservation | *P* value | *P* value |
| --- | --- | --- | --- | --- | --- |
| *Experimental samples* |  |  |  |  |  |
| Viable cells | 47.15 (43.8-48.65) |  | 43.3 (40.9-46.05)* | *na* | *0.05* |
| CD45^+^ cells | 93.3 (78.9-98.8) | 92.6 (84.05-96.3) | 97.1 (93.23-98.53) | *0.34* | *0.29* |
| CD11b^++^ cells | 9.34 (7.37-13.03) | 0.021 (0.01-0.06)* | 14.35 (11.06-16.78)* | *<0.001* | *0.01* |
| CD45^+^CD11b^+^ cells | 91.25 (86.95-92.63) |  | 86.25 (84.25-88.55)* | *na* | *0.01* |
| CD3^+^ cells | 72.75 (71.25-79.15) | 60.7 (58.9-64.2)* | 69.3 (67.83-75.13)* | *<0.001* | *0.02* |
| γδ TCR^+^ cells | 0.48 (0.2-1.43) | 1.95 (1.28-5.21)* | 0.38 (0.18-1.01) | *0.001* | *0.64* |
| CD161^+^ cells | 10.9 (9.13-11.7) | 4.27 (1.76-7.56)* | 11.3 (9.70-12.73) | *<0.001* | *0.17* |
| CD4^+^ cells | 87.1 (72.95-89.53) | 47.6 (33.7-53.55)* | 82.35 (74.23-87) | *<0.001* | *0.30* |
| CD8^+^ cells | 10.37 (7.38-19.28) | 13.9 (10.4-21.15) | 13.5 (9.58-20.58) | *0.30* | *0.34* |
| MAIT cells | 6.38 (1.41-8.83) | 1.39 (0.59-2.23)* | 5.71 (1.39-7.29) | *0.007* | *0.37* |
| Vα7.2^+^ cells | 16.75 (11.48-23.2) |  | 13.55 (10.48-21.63) | *na* | *0.34* |
| B cells | 51.25 (42.83-55.2) |  | 53.4 (45.2-58.8) | *na* | *0.40* |
| NK cells | 32.5 (21.63-41.15) | 3.64 (2.73-4.69)* | 26.75 (16.85-36.9) | *<0.001* | *0.27* |
| Classical monocytes | 1.12 (0.80-3.17) | 7.75 (5.06-13.2)* | 1.87 (0.82-3.30) | *<0.001* | *0.76* |
| Non-classical monocytes | 58.6 (55.93) | 0.69 (0.29-1.35)* | 60.4 (57.18-63.6) | *<0.001* | *0.55* |
| CD69^+^ | 0.98 (0.46-1.87) | 0.02 (0.01-0.05)* | 0.97 (0.01-1.53) | *<0.001* | *0.46* |
| HLA-DR^+^ | 2.21 (1.75-2.71) | 0.91 (0.55-1.17)* | 2.22 (1.53-2.83) | *<0.001* | *0.86* |
| GFAP^+^ astrocytes | 1.17 (0.60-2.32) | 1.51 (0.65-2.36) | 1.39 (0.81-5.15) | *0.84* | *0.28* |

Note: Transfix data excluded for CD45^+^CD11b^+^, Vα7.2^+^ and B cells. Values reported as median (interquartile range). Abbreviations: CD, cluster of differentiation; GFAP, glial fibrillary acidic protein; HLA-DR, human leukocyte antigen-DR; MAIT, mucosal associated invariant T cell; NK, natural killer; TCR, T cell receptor. Statistically significant results compared to the Fresh method are illustrated with an asterisk (*, p<0.05).

**Supplementary Table 4: Duration of storage comparison of cell percentages between Fresh vs Transfix methods.**

|  | **24 hour** | **1 week** | **2 week** |
| --- | --- | --- | --- |
| **CD45^+^ cells** |  |  |  |
| **CD11b^++^ cells** |  |  |  |
| **CD45^+^CD11b^+^ cells** |  | X | X |
| **CD3^+^ cells** |  |  |  |
| **γδ TCR^+^ cells** |  |  |  |
| **CD161^+^ cells** |  |  |  |
| **CD4^+^ cells** |  |  |  |
| **CD8^+^ cells** |  |  |  |
| **MAIT cells** |  |  |  |
| **Vα7.2^+^ cells** |  | X | X |
| **B cells** |  | X | X |
| **NK cells** |  |  |  |
| **Classical monocytes** |  |  |  |
| **Non-classical monocytes** |  |  |  |
| **HLA-DR^+^** |  |  |  |
| **GFAP^+^ astrocytes** |  |  |  |

Cell percentages were compared between fresh CSF and Transfix-treated CSF (n=3) stored for 24 hours, 1 week and 2 weeks. Green boxes signify no statistically significant difference between methods, whereas red boxes signify a statistically significant difference. Boxes demarcated with “X” represent cell populations that could not be accurately distinguished. Statistical significance was set at 0.05. CD, cluster of differentiation; HLA-DR, human leukocyte antigen-DR; GFAP, glial fibrillary acidic protein; TCR, T-cell receptor.

**Supplementary Table 5: Duration of storage comparison of cell percentages between Fresh vs Transfix methods**

|  | Fresh | 24 hour | 1 week | 2 week |
| --- | --- | --- | --- | --- |
| CD45^+^ cells |  | *0.05* |  | *0.05* |
| CD11b^++^ cells |  | *0.046* | *0.046* | *0.046* |
| CD45^+^CD11b^+^ cells |  | *0.046* | X | X |
| CD3^+^ cells |  |  | *0.05* | *0.05* |
| γδ TCR^+^ cells |  |  |  |  |
| CD161^+^ cells |  | *0.046* | *0.05* | *0.05* |
| CD4^+^ cells |  |  | *0.046* | *0.046* |
| CD8^+^ cells |  |  |  |  |
| MAIT cells |  | *0.05* |  |  |
| Vα7.2^+^ cells |  | *0.046* | X | X |
| B cells |  | *0.05* | X | X |
| NK cells |  | *0.05* | *0.05* | *0.05* |
| Classical monocytes |  | *0.05* | *0.05* |  |
| Non-classical monocytes |  |  | *0.05* | *0.05* |
| HLA-DR^+^ |  | *0.05* | *0.046* | *0.046* |
| GFAP^+^ astrocytes |  |  |  |  |

Note: *P* values are indicated where differences were statistically significant as compared to Fresh. Boxes demarcated with “X” refer to populations that could not be clearly distinguished during flow cytometric gating, and therefore were excluded from analyses.


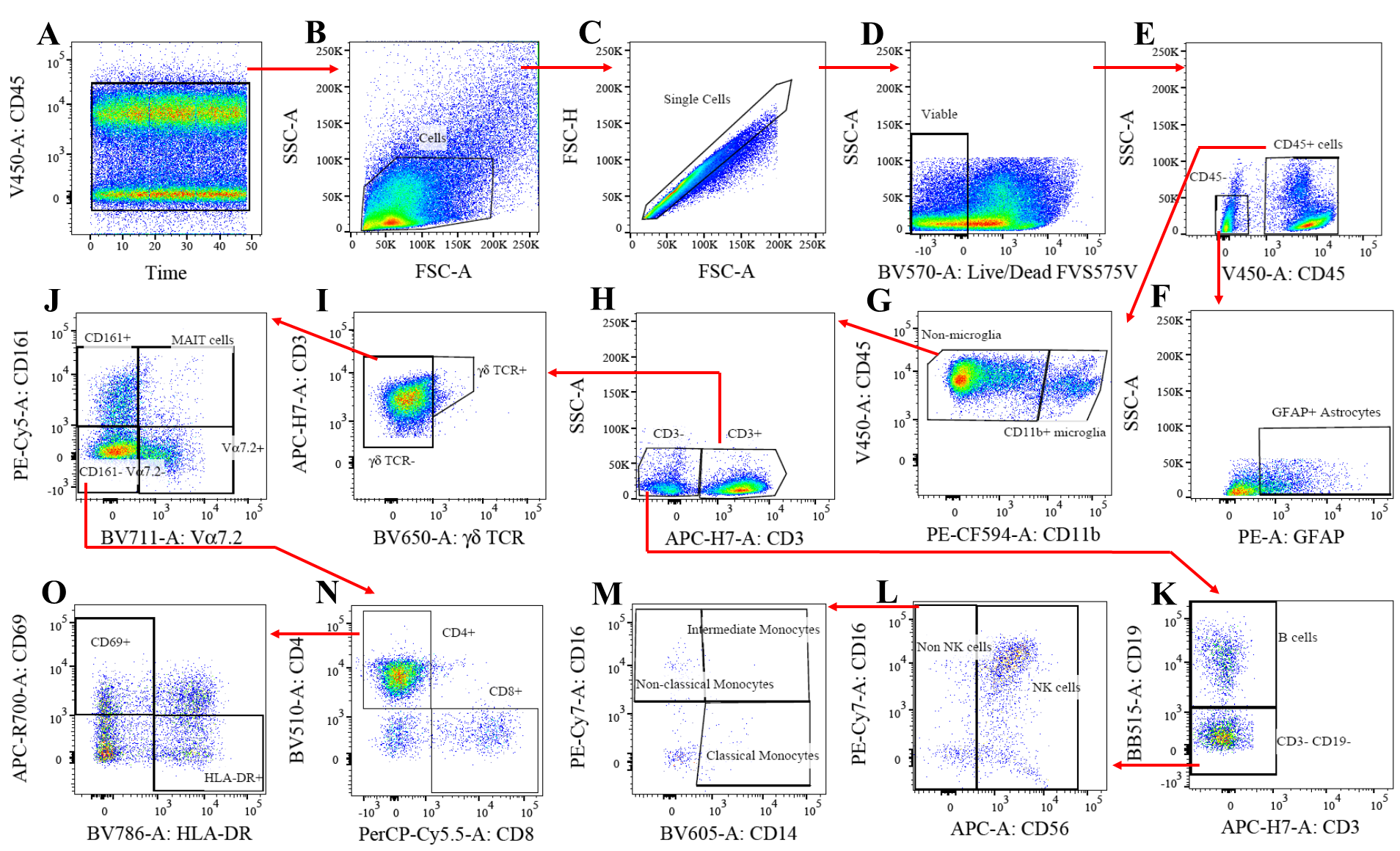


**Supplementary Figure 1: Gating strategy of patient sample.** Uniform fluorescence was selected with the “time” gate, cells were identified with side scatter-area (SSC-A) and forward scatter-area (FSC-A), single- and viable cells were selected by doublet and dye exclusion, respectively. Leukocytes (CD45^+^), T cells (CD3^+^), B cells (CD19^+^), NK cells (CD16^+^ CD56^+^), and sub-populations of monocytes (CD14^+^ & CD16^+^) were identified. Activation was assessed by HLA-DR and CD69 expression.

**Supplementary Figure 2: Gating strategy of experimental sample.** Uniform fluorescence was selected with the “time” gate, cells were identified with side scatter-area (SSC-A) and forward scatter-area (FSC-A), single- and viable cells were selected by doublet and dye exclusion, respectively. Leukocytes (CD45^+^), T cells (CD3^+^), B cells (CD19^+^), NK cells (CD16^+^ CD56^+^), and sub-populations of monocytes (CD14^+^ & CD16^+^) were identified. Activation was assessed by HLA-DR and CD69 expression.


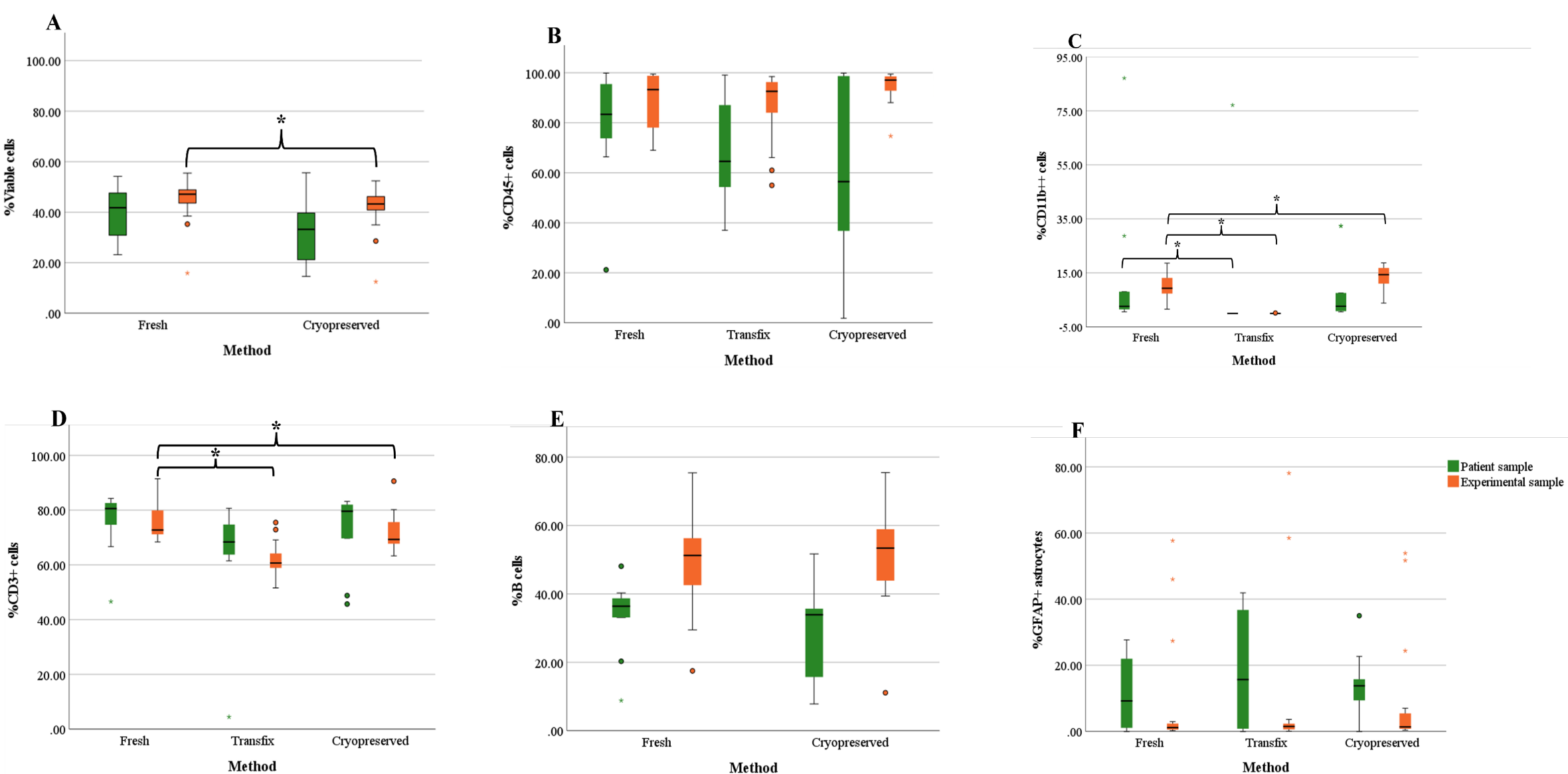


**Supplementary Figure 3: Boxplots comparing cell percentages by method in patient & experimental samples. (A)** %viable cells **(B)** %CD45^+^ cells **(C**) %CD11b^++^ **(D)** %CD3^+^ **(E**) %B cells **(F)** %GFAP^+^ astrocytes. Significance (p<0.05) indicated by the asterisk (*).


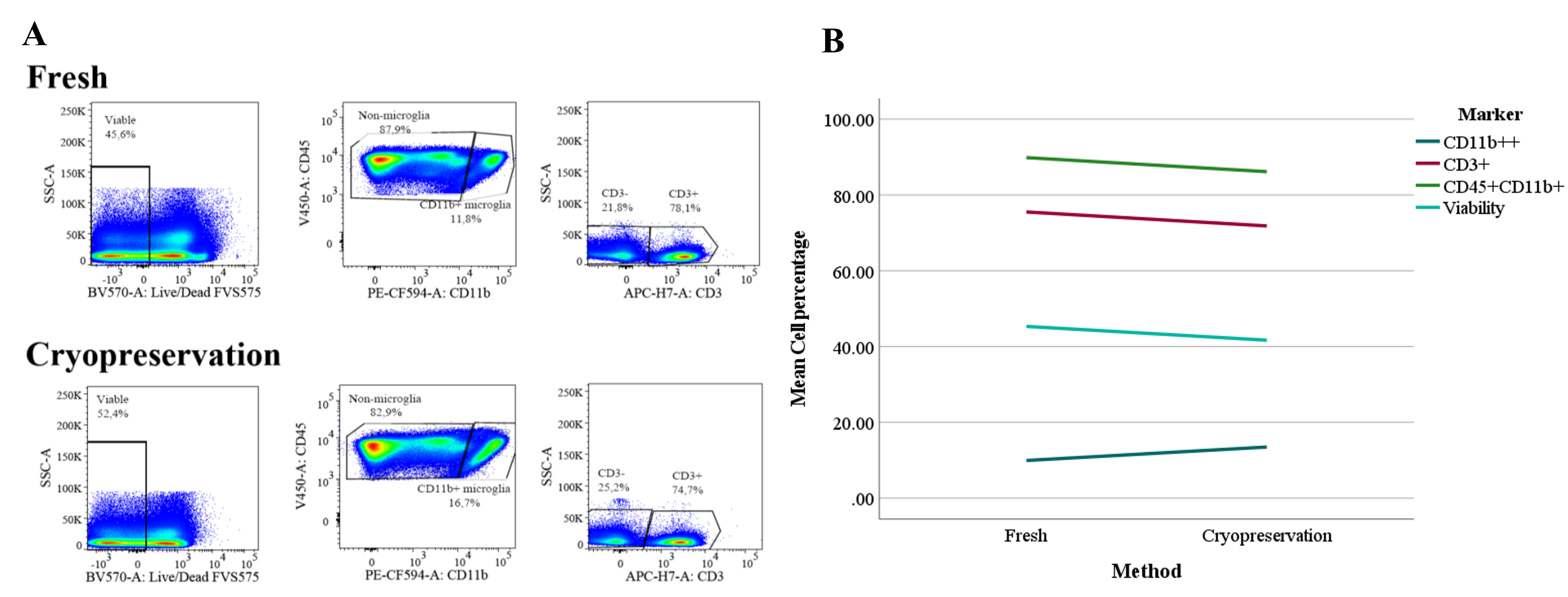


**Supplementary Figure 4A & B:** Example of varying cell percentage differences observed in Fresh versus Cryopreservation methods for experimental samples. CD45^+^CD11b^+^ (non-microglia) and CD3^+^ cell percentages were higher in the Fresh method. Whereas cell viability and CD11b^++^ (microglia) percentages were higher in Cryopreservation method in this example. Data from same sample.
